# Negative mood invites psychotic false perception in dementia

**DOI:** 10.1371/journal.pone.0197968

**Published:** 2018-06-01

**Authors:** Hiroyuki Watanabe, Yoshiyuki Nishio, Yasuyuki Mamiya, Wataru Narita, Osamu Iizuka, Toru Baba, Atsushi Takeda, Tatsuo Shimomura, Etsuro Mori

**Affiliations:** 1 Department of Behavioral Neurology and Cognitive Neuroscience, Tohoku University Graduate School of Medicine, Sendai, Japan; 2 Department of Neurology, Sendai Nishitaga National Hospital, Sendai, Japan; 3 Department of Rehabilitation Medicine, Akita Prefectural Centre of Rehabilitation and Psychiatric Medicine, Daisen, Japan; 4 Department of Behavioral Neurology and Neuropsychiatry, Osaka University United Graduate School of Child Development, Suita, Japan; Chiba Daigaku, JAPAN

## Abstract

**Background:**

There is increasing evidence for predictive coding theories of psychosis, which state that hallucinations arise from abnormal perceptual priors or biases. However, psychological processes that foster abnormal priors/biases in patients suffering hallucinations have been largely unexplored. The widely recognized relationship between affective disorders and psychosis suggests a role for mood and emotion.

**Methods:**

Thirty-six patients with dementia with Lewy bodies (DLB), a representative condition associated with psychosis of neurological origin, and 12 patients with Alzheimer’s disease (AD) were enrolled. After an experimental mood induction, the participants underwent the pareidolia test, in which visual hallucination-like illusions were evoked and measured.

**Results:**

In DLB patients, the number of pareidolic illusions was doubled under negative mood compared to that under neutral mood. In AD patients, there was no significant difference in the number of pareidolic responses between negative and neutral mood conditions. A signal detection theory analysis demonstrated that the observed affective modulation of pareidolic illusions was mediated through heightened perceptual bias, not sensory deterioration.

**Conclusions:**

The current findings demonstrated that abnormal perceptual priors in psychotic false perception have an affective nature, which we suggest are a type of cognitive feeling that arises in association with perception and cognition.

## Introduction

In predictive coding theories, perception is not a faithful copying of objects in the external world but rather an active inference process based upon priors and sensory evidence. According to this view, false perceptions in psychosis, e.g., hallucinations and illusions, can be understood as abnormalities in priors [1]. Several experiments, in which priors were given as explicit knowledge or implicitly learned associations directly relating to behavioral goals in tasks at hand, have supported this ‘psychosis-as-abnormal-priors’ hypothesis [2–4]. In contrast, patients suffering psychosis spontaneously foster abnormal perceptual priors/biases through a context not directly linked to current behavioral goals. However, the psychological underpinnings of the abnormal perceptual priors in psychosis have been largely unexplored.

Mood or emotional states have a substantial impact on simple perceptual decision making in everyday life. When you are alone and anxious in a dark room, you may clearly hear a small noise that is ordinarily unnoticed. This phenomenon is probably not because negative mood improves or deteriorates your sensation itself but because anxiety biases your perception. Just as in healthy people, a relationship between negative mood, e.g., depression and anxiety, and auditory hallucinations has been implicated in patients with psychosis [5], suggesting that mood may play an important role in the development of abnormal perceptual priors in psychosis.

Psychosis is associated not only with mental illness but also with neurological disorders. Dementia with Lewy bodies (DLB), which is the second most common degenerative dementia after Alzheimer’s disease (AD), is a classic example of psychosis of neurological origin, in which visual hallucinations are one of the core symptoms [6]. We recently developed a simple visual task that evokes and measures visual hallucination-like illusions for DLB and other neurological disorders, called the pareidolia test [7,8]. In the present study, we utilized an experimental mood induction procedure and the pareidolia test to investigate the role of mood in perceptual priors/biases associated with psychotic false perception.

## Material and methods

### 2.1. Participants

Thirty-six consecutive patients with DLB participated in the study. Twelve patients with AD, who were matched to the DLB patients based on age, sex, education, visual acuity and Mini-Mental State Examination (MMSE) score, were recruited as disease control subjects. All patients underwent an examination by experienced behavioral neurologists, magnetic resonance imaging of the brain and routine laboratory investigations. DLB and AD were diagnosed according to the consensus diagnostic criteria [6,9]. At the time of examination, 15 patients with DLB and 8 patients with AD were treated with donepezil, and 9 patients with DLB were treated with levodopa. The patients were not taking antipsychotics or antidepressants. The demographic and clinical characteristics of the participants are summarized in **Table 1**.

**Table 1:** Demographic characteristics of the participants.

	DLB (n = 36)	AD (n = 12)	p-values
Age, years ‡	79.8 (7.4)	78.8 (6.2)	0.707
Sex (female/male) †	22 / 14	10 / 2	0.157
Education, years ‡	9.3 (2.4)	9.2 (2.5)	0.892
Visual acuity ‡	0.6 (0.1)	0.6 (0.2)	0.113
Neuropsychology ‡			
MMSE [30]	16.5 (4.3)	17.8 (3.8)	0.352
ACE-R Total [100]	49.2 (19.0)	54.7 (14.4)	0.381
Attention/Orientation [18]	10.0 (3.6)	11.3(3.7)	0.302
Memory [26]	8.5 (5.1)	7.4 (2.5)	0.477
Verbal fluency [14]	5.0 (4.0)	7.1 (3.2)	0.115
Language [26]	17.6 (5.4)	17.3 (4.6)	0.858
***Visuospatial [16]***	***8*.*1 (4*.*7)***	***11*.*7 (2*.*8)***	***0*.*021*** ^***a***^
Shape detection [20]	17.5 (2.3)	18.5 (1.4)	0.177
Position discrimination [20]	17.5 (2.8)	19.0 (1.9)	0.087
Face recognition [30]	23.7 (3.3)	24.9 (2.8)	0.251
NPI §			
Persecutory delusions	1.0 (4.0)	0.0 (2.5)	0.383
***Delusional misidentifications***	***1*.*5 (4*.*0)***	***0*.*0 (0*.*0)***	***0*.*032*** ^***b***^
***Hallucinations***	***2*.*0 (3*.*0)***	***0*.*0 (0*.*0)***	***< 0*.*001*** ^***b***^
Agitation/aggression	0.0 (4.0)	0.5 (4.5)	0.613
Depression	0.0 (3.0)	0.0 (4.5)	0.869
Anxiety	0.0 (4.0)	0.0 (0.3)	0.209
Euphoria	0.0 (0.0)	0.0 (0.0)	0.406
Apathy	2.5 (8.0)	0.0 (3.3)	0.13
Disinhibition	0.0 (0.0)	0.0 (0.3)	0.75
Irritability/lability	0.0 (4.0)	0.0 (3.8)	0.784
Aberrant motor behavior	0.0 (4.0)	0.0 (0.0)	0.193
Fluctuations in cognition	2.5 (8.0)	0.0 (3.3)	0.254

The NPI scores are presented as the medians (interquartile range). The other scores are presented as the means (standard deviations). ACE-R scores were not available for one patient with DLB.

†Chi-squared test

‡Two-sample t-test

§Mann-Whitney U test.

^a^ DLB < AD (p < 0.05)

^b^ AD < DLB (p < 0.05).

MMSE, Mini-Mental State Examination; ACE-R, Addenbrooke's Cognitive Examination-Revised; NPI, Neuropsychiatric Inventory.

All procedures in this study were approved by the ethics committee of the Tohoku University Graduate School of Medicine. All participants and caregivers provided written informed consent after receiving a detailed explanation of the study.

### 2.2. Background neuropsychological and behavioral assessments

Addenbrooke’s Cognitive Examination-Revised (ACE-R) provides domain scores for attention/orientation, memory, verbal fluency, language, and visuospatial function and was used to assess various cognitive domains in this study [10,11]. We also used the Shape Detection Screening and Position Discrimination subtests of the Visual Object and Space Perception battery [12] and the Face Recognition subtests (face-to-face matching of unknown faces, same/different judgement of unknown faces in different views and gender and age judgements of unknown faces) of the Visual Perception Test for Agnosia to assess visuoperceptual and visuospatial functions [13]. The MMSE and ACE-R total scores were used as measures of global cognitive function.

The Neuropsychiatric Inventory (NPI) was administered to the patients’ caregivers [14]. The original NPI consists of the following 10 behavioral domains: delusions, hallucinations, depression/dysphoria, anxiety, agitation/aggression, euphoria, disinhibition, irritability/lability, apathy, and aberrant motor behavior. For the delusion domain, we separately assessed persecutory delusions and delusional misidentifications [15]. Fluctuation in cognition was assessed using the Cognitive Fluctuation Inventory [16].

### 2.3. Experimental investigations

**Fig 1:**
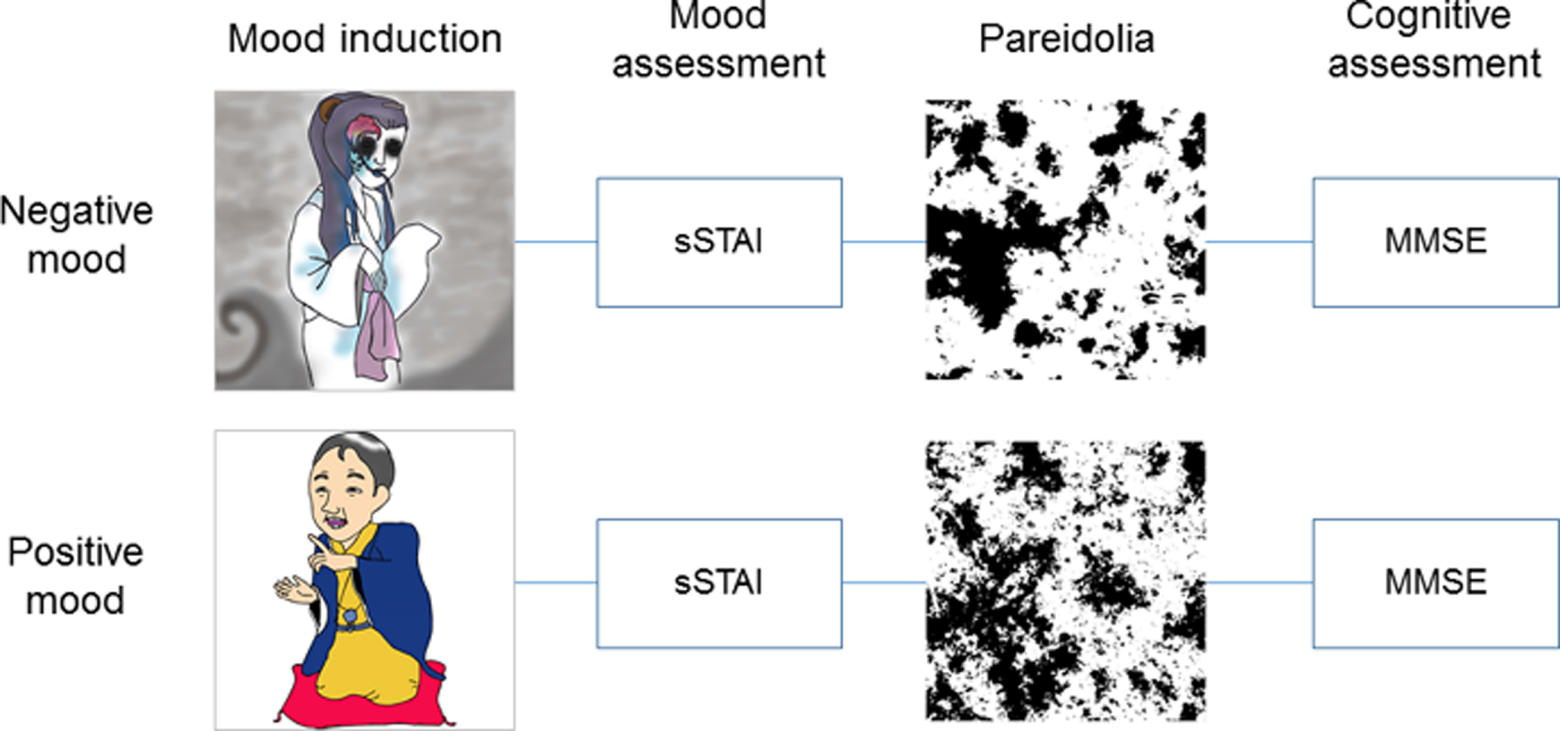
Outline of the experiment. sSTAI, short form of the State-Trait Anxiety Inventory; MMSE, Mini-Mental State Examination.

The experimental procedures are outlined in **Fig 1**. All participants underwent 3 experimental sessions, each of which was associated with positive, negative or neutral mood states. Each experimental session consisted of the following 4 phases: mood induction, mood assessment, the pareidolia test and cognitive testing. Patients’ mood states were defined by experimental manipulations in the mood induction phase, and all other procedures were identical across the 3 sessions. The order of the positive mood, negative mood and neutral mood sessions was counterbalanced across participants. The between-session intervals ranged from 5 to 63 days.

#### 2.3.1. Mood induction phase

To induce each target mood (positive, negative or neutral), a recorded story and instrumental music were presented to the participants at the beginning of each experimental session. A classic Japanese comedy story (落語rakugo) and happy music were used as mood inducers in the positive mood session. In the negative mood session, a classic Japanese horror story (怪談 kaidan) and gloomy music were used. The durations of the story and music presentations were similar between the negative and positive mood conditions (148 seconds in the negative mood condition and 129 seconds in the positive mood conditions). In the neutral mood session, neither a story nor music were presented.

#### 2.3.2. Mood assessment phase

To assess the effects of the mood induction procedures on the participants’ mood, we administered the short form of the State-Trait Anxiety Inventory (sSTAI) immediately after the mood induction phase [17]. The sSTAI is a self-report questionnaire consisting of 6 items (calm, tense, upset, relaxed, content, and worried), each scored on a 4-point, forced-choice Likert-type response scale, and the total scores range from 6 to 24, with higher scores indicating greater levels of anxiety.

#### 2.3.3. Pareidolia test phase

We used the noise pareidolia test as a measure of visual hallucination-like false perception [7,15]. To increase the sensitivity and quantitative capability of the test, we used the current version of the test, which has a larger number of stimuli than the original version. Accordingly, the test contained 80 black-and-white visual noise images; half of the images had a spatial frequency of 1/f^2.5^, and the other half had a spatial frequency of 1/f^3^. Black-and-white images of human faces were embedded in 16 of the 80 images. The participants were requested to state whether a face was present and point to the location where they observed a face. Each picture was presented for a maximum of 60 seconds, and no feedback was provided to the subjects, regardless of whether the responses were correct. Immediately before the beginning of the test, a detailed explanation was provided, and 7 training trials were administered. Participants’ responses were classified into the two following types: (1) pareidolic responses, in which subjects incorrectly identified faces in the stimuli, and (2) correct responses, in which subjects correctly responded with “nothing exists” to stimuli that did not contain faces or correctly detected an embedded face in the stimuli. When subjects responded with such comments as “It looks like a face”, we asked the subjects whether there was an actual face in the noise stimuli or whether the subject saw something that simply looked similar to a face. Only the former response was regarded as pareidolic.

#### 2.3.4. Cognitive testing phase

Induced mood may modulate not only visual perception but also other aspects of cognition. To examine this possibility, we administered the MMSE to participants immediately after the pareidolia test. To avoid learning effects in the word-recall task, different word lists were used for each experimental session.

### 2.4. Signal detection theory analysis

In our previous study, we suggested that pareidolic illusions arise from a heightened endogenous bias to see faces rather than from a visuoperceptual impairment in patients with DLB [15]. To test whether emotional modulation of pareidolic illusions was mediated thorough changes in bias or changes in visuoperceptual ability, we performed a signal detection theory (SDT) analysis. Because the distribution of the data in the pareidolia test was severely skewed, we used the *d*_*e*_*´* (discrimination accuracy) and *C*_*e*_ (criterion or bias) SDT indices instead of the conventional *d´* and *C* indices, respectively. These SDT indices were calculated with the following formula:

*d*_*e*_*'* = {2 / (1 + *s*)}z(HR)—*s*z(FAR),

*C*_*e*_ = —{2*s* / (1 + *s*)^2^}{z(HR) + z(FAR)}

where z(HR) is a z-transformed rate of hits (correct response rate for the stimuli that contained a face), z(FAR) is a z-transformed rate of false alarms (pareidolic response rate for the stimuli that did not contain a face), and *s* is the slope of the linear regression line for the z(HR) and z(FAR) values [18]. Data with floor or ceiling effects were adjusted as follows: rates of 0 and 1 were replaced with 0.5/n and (n-0.5)/n, respectively, where n is the number of trials [19].

### 2.5. Statistical analyses

The effects of induced mood and diagnosis on the sSTAI and pareidolia test scores were analyze for DLB patients using a linear mixed-effects model with a random intercept. The fixed effects were mood category (neutral, negative or positive) and diagnosis (DLB vs AD), and the random effect was participant with the mood categories nested under each participant. Pre-determined pairwise contrasts for the mood categories, “neutral mood vs positive mood” and “neutral mood vs negative mood”, were included in the model. The impact of induced mood on the SDT indices was only investigated in the DLB group. This analysis was performed with a linear mixed-effects model analysis in which the fixed and random effects were mood category and participant, respectively, and the mood categories were nested under participants. The “neutral mood vs positive mood” and “neutral mood vs negative mood” contrasts were included in the model. The *nlme* package for R was used for the linear mixed-effects model analyzes [20].

To identify factors that predict the impacts of experimentally induced moods on pareidolic illusions, we performed a multiple linear regression analysis with the following variables:

Dependent variable: (PRneg -PRneut) / (PRneut + 0.5),

where PRneg and PRneut stand for the number of pareidolic responses under the negative mood condition and that under the neutral mood condition, respectively. This variable represents the amount of affective modulation of pareidolic illusions.

Independent variables:

(1) sSTAIneg—sSTAIneut;
(2) NPI hallucination domain score;
(3) NPI depression domain score;
(4) NPI anxiety domain score;
(5) NPI euphoria domain score;
(6) ACE-R visuospatial score;
and (7) ACE-R memory score,

where sSTAIneg and sSTAIneut are the sSTAI score under the negative mood condition and that under the neutral mood condition, respectively. The first variable represents the intensity of the experimentally induced negative mood.

## Results

### 3.1. Effects of the mood induction procedure on mood

**Fig 2:**
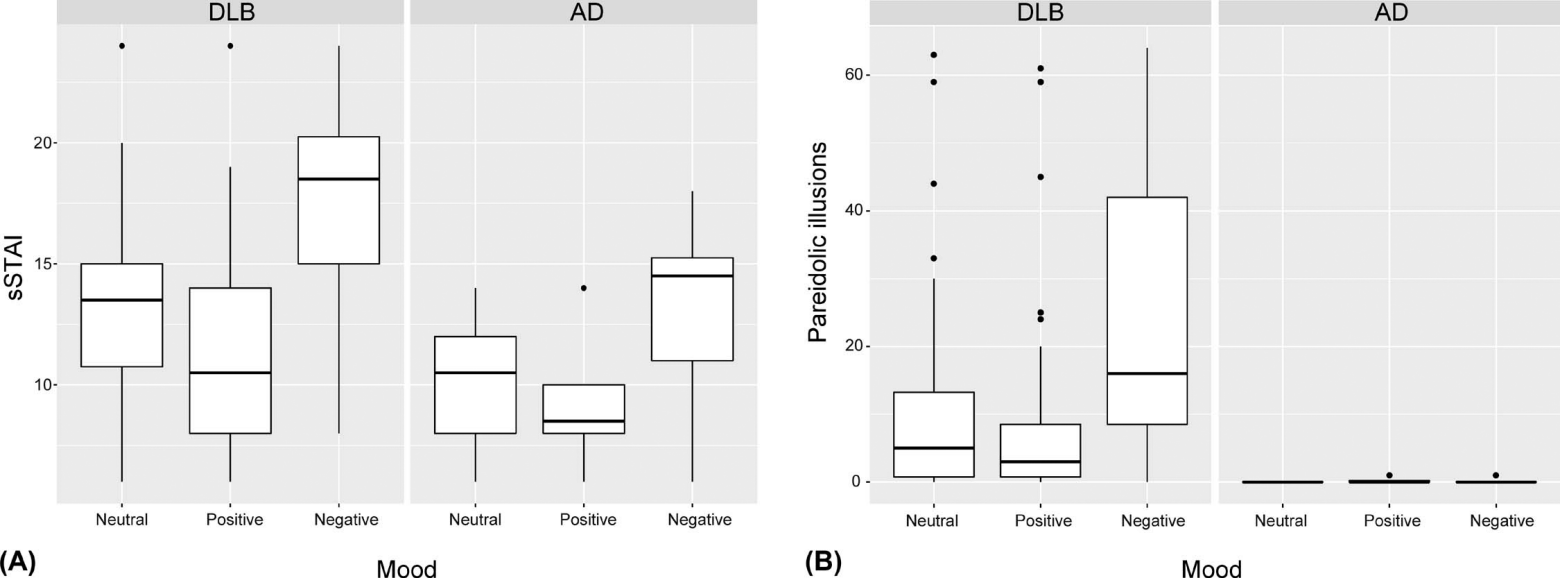
Effects of the mood induction procedure on mood and pareidolic illusions. (A) In both the DLB and AD groups, the sSTAI scores were highest under the negative mood condition and lowest under the positive mood condition, which indicates that the target moods were successfully induced by the experimental manipulations. DLB, dementia with Lewy body disease; AD, Alzheimer's disease; sSTAI, short form of the State-Trait Anxiety Inventory. (B) In the DLB group, the numbers of pareidolic responses were significantly larger under the negative mood condition (24.1 ± 20.5) than those under the neutral mood condition (11.2 ± 16.1) and were similar between the positive (9.4 ± 15.4) and neutral mood conditions. There were no significant differences in the number of pareidolic responses across the 3 mood conditions in the AD group.

The sSTAI scores under the 3 mood conditions are shown in **Fig 2A**. In the DLB group, the sSTAI scores (mean ± standard deviation) were 12.9 ± 4.2, 11.3 ± 4.0 and 17.3 ± 4.4 under the neutral, positive and negative mood conditions, respectively. In the AD group, the sSTAI scores were 10.1 ± 2.6, 8.9 ± 1.9 and 13.4 ± 3.4 under the neutral, positive and negative conditions, respectively. The mixed-effects model analysis revealed significant mood effects between the neutral and negative conditions (t(92) = 7.94, p < 0.001) and between the neutral and positive conditions (t(92) = -2.95, p = 0.004) and a significant effect of the diagnosis (t(46) = -2.13, p = 0.039). No significant interactions between mood and diagnosis were observed (t(92) = -0.97, p = 0.33 for the neutral-negative mood contrast × the diagnosis; t(92) = 0.42, p = 0.67 for the neutral-positive mood contrast × the diagnosis). Thus, the mood induction procedures employed here produced the desired effects on the moods of the participants.

### 3.2. Effects of mood on pareidolic illusions

The numbers of pareidolic responses on the pareidolia test under the 3 mood conditions are shown in **Fig 2B**. In the DLB group, there were twice as many pareidolic responses under the negative mood condition as there were under the neutral and positive conditions (11.2 ± 16.1, 9.4 ± 15.4 and 24.1 ± 20.5 under the neutral, positive and negative mood conditions, respectively). Thirty-two of the 36 DLB patients produced 1 or more pareidolic responses under the negative mood condition than under the neutral mood condition. In AD patients, the numbers of pareidolic responses did not significantly differ across the three mood conditions (0.0 ± 0.0, 0.3 ± 0.4 and 0.1 ± 0.3 under the neutral, positive and negative mood conditions, respectively). Only 1 of 12 AD patients produced 1 or more pareidolic responses under the negative mood condition than under the neutral mood condition. The mixed-effects model analysis showed a significant mood effect between the neutral and negative conditions (t(92) = 6.49, p < 0.001), a significant effect of the diagnosis (t(46) = -2.17, p = 0.035) and a significant interaction between the neutral-negative mood contrast × the diagnosis (t(92) = -3.22, p = 0.002). Neither a significant effect of the neutral-positive mood contrast (t(92) = -0.91, p = 0.37) nor a significant interaction between the neutral-positive mood contrast × the diagnosis (t(92) = 0.52, p = 0.61) was observed. In summary, pareidolic illusions were increased by negative mood in patients with DLB but not in those with AD.

### 3.3. Effects of mood on cognitive function

In the DLB group, the MMSE scores were 16.4 ± 4.6, 15.8 ± 4.9 and 16.9 ± 4.5 under the neutral, positive and negative mood conditions, respectively. In the AD group, the MMSE scores were 17.8 ± 3.8, 16.6 ± 4.4 and 17.3 ± 3.7 under the neutral, positive, and negative mood conditions, respectively. We did not observe any significant effects of mood (t(92) = 1.07, p = 0.29 for the neutral-negative contrast; t(92) = -1.68, p = 0.10 for the neutral-positive contrast) or diagnosis (t(46) = 0.91, p = 0.37) on the MMSE scores. In addition, no interactions between the mood contrasts and the diagnosis were observed (t(92) = -1.24, p = 0.22 for the neutral-negative contrast × the diagnosis; t(92) = -0.67, p = 0.50 for the neutral-positive contrast × the diagnosis).

### 3.4. Results of the SDT analysis

**Fig 3:**
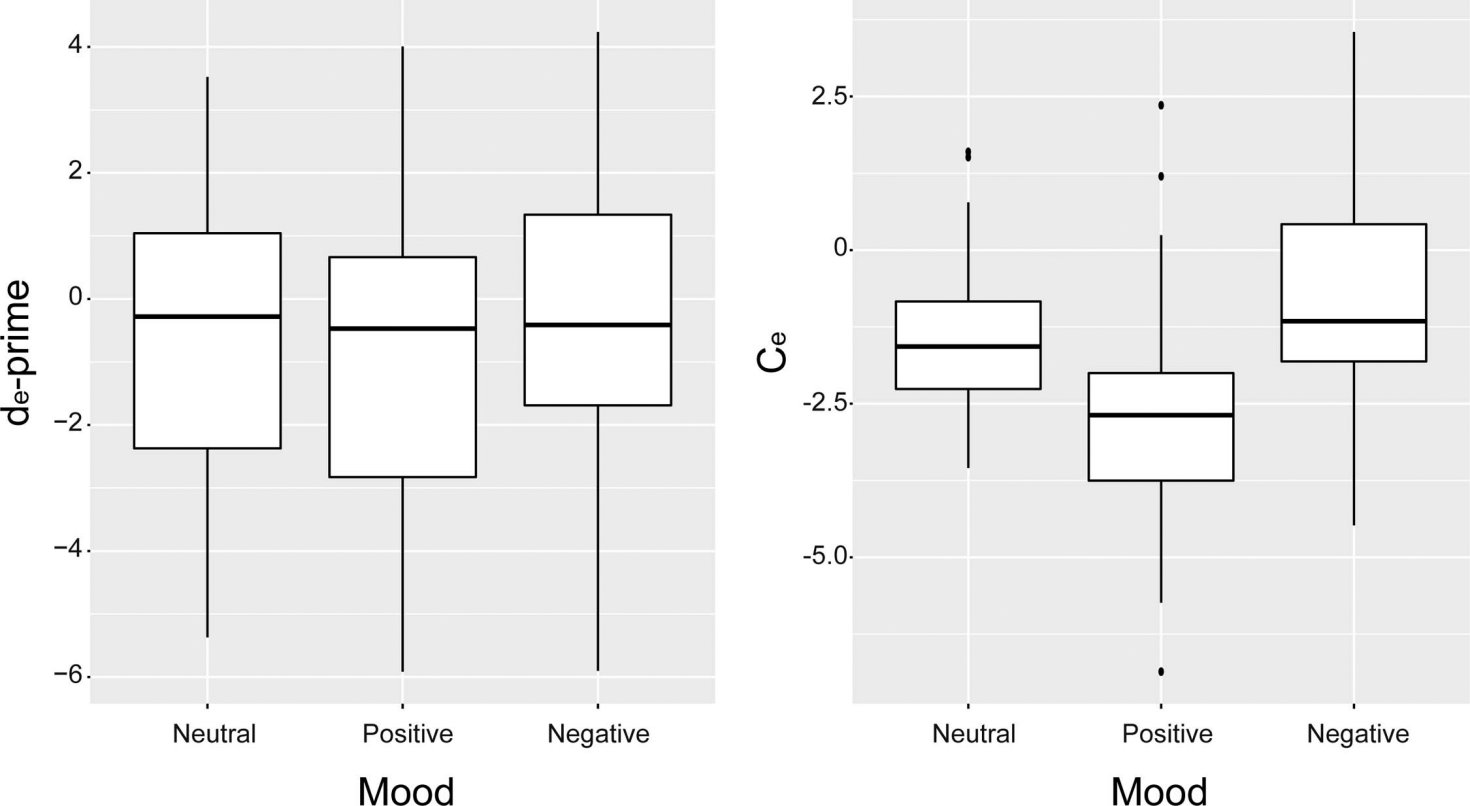
Signal detection theory analysis of the pareidolia test for DLB patients. (Left) No significant differences were observed across the 3 mood conditions in *d*_*e*_*'* (discrimination ability). (Right) *Ce* (criterion/bias) was significantly modulated by the induced mood and was highest under the negative mood condition and lowest under the positive mood condition. DLB, dementia with Lewy body disease.

The SDT results for the patients with DLB are shown in **Fig 3**. The *d*_*e*_*'* values were -0.8 ± 2.5, -0.9 ± 2.6 and -0.5 ± 2.5 under the neutral, positive and negative mood conditions, respectively. The *C*_*e*_ values were -1.4 ± 1.2, -2.7 ± 1.8 and -0.8 ± 1.7 under the neutral, positive and negative mood conditions, respectively. No significant mood effects on *d*_*e*_*'* (t(70) = 0.94, p = 0.35 for the neutral-negative contrast; t(70) = -0.42, p = 0.67 for the neutral-positive contrast) were observed. Both the induced negative and positive moods had significant effects on *C*_*e*_ (t(70) = 2.78, p = 0.007; t(70) = -6.19, p < 0.001, respectively). In summary, *C*_*e*_ (criterion or bias) but not *d*_*e*_*'* (discrimination ability) significantly changed depending on the mood states.

### 3.5. Results of the regression analysis

The results are summarized in **Table 2**. More severe clinical hallucinations (NPI hallucination domain score) predicted higher degrees of affective modulation of pareidolic illusions in patients with DLB.

**Table 2:** Results of the multiple linear regression analysis.

Factors	R^2^	B	SE-B	p-values
	0.177			
sSTAI		0.485	0.818	0.559
***Hallucinations***		***3*.*586***	***1*.*057***	***0*.*002***
Depression		-2.451	1.348	0.080
Anxiety		0.068	0.913	0.941
Euphoria		-1.186	2.709	0.665
Visuospatial		0.839	0.665	0.218
Memory		-0.003	0.604	0.996

R^2^, coefficient of determination; B, regression coefficient; SE-B, standard error of the regression coefficient.

## Discussion

The present study demonstrated that hallucination-like visual illusions (pareidolic illusions) significantly increased under negative mood compared to those under neutral or positive mood in neurological patients with psychosis. The SDT analysis demonstrated that the moods had significant impacts on *C*_*e*_ (criterion or bias) but not on *d*_*e*_*'* (discrimination ability), indicating that the observed modulations of pareidolic illusions by moods were mediated by heightened abnormal perceptual bias rather than sensory deterioration. These findings suggest that abnormal perceptual priors/biases in psychotic false perception have an affective nature that is based on implicit or unconscious psychological processes that are not directly linked to behavioral goals in tasks at hand.

In the present study, neither patients’ affective traits nor the intensity of the experimentally induced mood but only patients’ states of visual hallucinations effectively predicted the degree of affective modulation of pareidolic illusions, indicating that the abnormal perceptual priors associated with visual hallucinations and pareidolic illusions are modulated by mood but are not affective mood itself. In previous theories, subjective experiences associated with perceptual and cognitive processes that are distinct from emotions/moods have been termed cognitive feelings [21]. Primary examples of cognitive feelings include the feeling of familiarity in object recognition, the feeling of knowing in episodic memory retrieval and the sense (feeling) of agency in self-consciousness. These cognitive feelings are used as information in various types of decision making, easily imbued with affective valance and modulated by current emotions and moods [22]. We suggest that abnormal perceptual priors associated with psychotic false perception can be conceptualized as this type of cognitive feeling. Capgras delusions are a good example of how an abnormal cognitive feeling leads to psychotic false perception. Patients suffering from Capgras delusions falsely identify a person close to them, such as their spouse or child, as someone else whose appearance is identical to that familiar person, indicating that intact perceptual recognition of physical features is not sufficient to correctly identify familiar persons. A seminal hypothesis by Ellis and Young postulates that person identification is based on two parallel processes, one of which is the conscious or overt cognitive process of visual face recognition and the other is the unconscious or covert affective process [23]. This hypothesis explains Capgras delusions as abnormalities in covert affective processing, i.e., the feeling of familiarity. Translating this framework into the terms of predictive coding theories, an abnormal prior (feeling of familiarity) with intact sensory evidence (conscious visual face recognition) leads to Capgras delusions.

We suggest that two empirical concepts in the phenomenology of psychosis may be relevant to the cognitive feelings associated with psychotic false perception. The first is an abnormal ‘feeling (sense) of presence’, which is a feeling or sensation that someone is nearby. An abnormal feeling of presence was first described in relation to schizophrenia by Jaspers and has recently been recognized as a common psychotic phenomenon in Parkinson’s disease and DLB [24,25]. Recent studies suggest that an abnormal feeling of presence is presumably a symptom of a predisposition to frank visual hallucinations [26,27]. In healthy people, a feeling of presence usually arises in association with spatial perception of external stimuli [21]. Under an anxious mood, however, people feel a presence even without external stimuli, which illustrates the affective property of the feeling of presence. The other key concept in the phenomenology of psychosis is ‘aberrant motivational salience’. Kapur proposed that the assignment of inappropriate salience and motivational significance to external objects and/or internal representations leads to hallucinations and delusions in psychosis [28]. This view is based on the observation that patients with schizophrenia often experience a greater awareness or keenness of certain percepts and ideas before the development of full-fledged psychosis. We suggest that an abnormally heightened feeling of presence and/or aberrant motivational salience may serve as an abnormal prior in the development of psychotic false perception.

In conclusion, the present study demonstrated that false perception was significantly exacerbated under negative mood in neurological patients with psychosis. We suggest that abnormal cognitive feelings, which are unconscious affective processes that arise in association with perception and cognition, serve as an abnormal prior in psychotic false perception. Further identification of psychological and biological properties of cognitive-affective interactions in psychosis would be beneficial for the future development of effective and safe treatments for psychosis.

## Supporting information

S1 Appendix Neuropsychological results of individual participants. (XLSX)
